# Overexpression of *MYB115*, *AAD2*, or *AAD3* in *Arabidopsis thaliana* seeds yields contrasting omega-7 contents

**DOI:** 10.1371/journal.pone.0192156

**Published:** 2018-01-30

**Authors:** Hasna Ettaki, Manuel Adrián Troncoso-Ponce, Alexandra To, Guillaume Barthole, Loïc Lepiniec, Sébastien Baud

**Affiliations:** 1 Institut Jean-Pierre Bourgin, Institut National de la Recherche Agronomique, AgroParisTech, Centre National de la Recherche Scientifique, Université Paris-Saclay, Versailles, France; 2 Sorbonne Universités, Génie Enzymatique et Cellulaire, Formation de Recherche en Evolution Centre National de la Recherche Scientifique 3580, Université de Technologie de Compiègne, Compiègne, France; 3 Université Paris-Sud, Université Paris-Saclay, Orsay, France; RIKEN Center for Sustainable Resource Science, JAPAN

## Abstract

Omega-7 monoenoic fatty acids (ω-7 FAs) are increasingly exploited both for their positive effects on health and for their industrial potential. Some plant species produce fruits or seeds with high amounts of ω-7 FAs. However, the low yields and poor agronomic properties of these plants preclude their commercial use. As an alternative, the metabolic engineering of oilseed crops for sustainable ω-7 FA production has been proposed. Two palmitoyl-ACP desaturases (PADs) catalyzing ω-7 FA biosynthesis were recently identified and characterized in *Arabidopsis thaliana*, together with MYB115 and MYB118, two transcription factors that positively control the expression of the corresponding PAD genes. In the present research, we examine the biotechnological potential of these new actors of ω-7 metabolism for the metabolic engineering of plant-based production of ω-7 FAs. We placed the PAD and MYB115 coding sequences under the control of a promoter strongly induced in seeds and evaluated these different constructs in *A*. *thaliana*. Seeds were obtained that exhibit ω-7 FA contents ranging from 10 to >50% of the total FAs, and these major compositional changes have no detrimental effect on seed germination.

## Introduction

Vegetable oils enriched in omega-7 (ω-7) monounsaturated fatty acids (FAs) such as palmitoleic acid (*cis*-ω-7 C16:1) and its elongation product vaccenic acid (*cis*-ω-7 C18:1) potentially have uses for a number of applications. First, they are attractive for biodiesel formulations since biodiesel containing FA methyl esters produced from oils rich in ω-7s has superior functional properties [1]. In addition, ω-7s from plant oils can offer the chemist promising starting material for catalytic conversions to renewable platform chemicals. Olefin metathesis constitutes a powerful tool for polymer chemistry, and ethenolytic metathesis of ω-7 FAs from plant oils could potentially provide a competitive source of 1-octene to make linear low-density polyethylene [2]. Finally, after the fast growth of ω-3 polyunsaturated FAs in the nutrition market [3], suppliers and marketers are today calling attention to ω-7 FAs since vegetable oils enriched in ω-7s have been ascribed a number of beneficial health properties. Palmitoleic acid in particular is considered a lipokine used by adipose tissues to communicate with distant organs and regulate systemic metabolic homeostasis [4]. Increasing evidence suggests that palmitoleic acid plays a key role in the physiopathology of insulin resistance in humans, increasing muscle response to insulin [5]. Aside from these nutritional functions, palmitoleic acid is attractive for nonfood uses in the skin care industry because of its antioxidant and antimicrobial properties [6].

Natural plant oils containing high levels of ω-7 FAs are infrequent. Foods and nutraceuticals enriched in ω-7s for health purpose are often sourced from sea buckthorn berries (*Hippophae rhamnoides*) that contain up to 50% ω-7s in pulp oil [7]. Seed oils enriched in ω-7 FAs have been described in milkweed (*Asclepias syriaca*; ∼25% ω-7s), macadamia (*Macadamia integrifolia*; ∼35% ω-7s), cat’s claw vine (*Doxantha unguis-cati*; ∼75% ω-7s), *Roureopsis obliquifoliata* (∼30% ω-7s), and *Entandrophragma cylindricum* (∼55% ω-7s), among others (phylofadb.bch.msu.edu). These plants exhibit low yields and poor agronomic properties. Oilseed crops contain very low amounts of ω-7 monounsaturated FAs. To a certain extent, rapeseed (*Brassica napus*) is an exception to this rule. In seeds of *B*. *napus*, as in seeds of its close relative *Arabidopsis thaliana*, the proportion of ω-7s accounts only for a small percentage of total seed FAs [8], but ω-7 FAs are highly concentrated in the endosperm, a seed compartment of reduced size that comprises a single cell layer surrounding the embryo in mature dry seeds [9,10]. Vaccenic acid and paullinic acid (*cis*-ω-7 C20:1) thus represent more than 20 mol% of total FAs in the endosperm fraction of *A*. *thaliana* seeds, and approximately 35 mol% in rapeseed [11].

Production of saturated FAs is performed in a stepwise manner, in plastids, by the FA synthase of type II. Saturated acyl chains, bound to acyl-carrier proteins (ACPs), can then be desaturated by stromal soluble acyl-ACP desaturases (AADs) to form *cis*-monoenes. Depending on both their substrate specificity and their regiospecificity, activities of AAD isoforms can give rise to a range of *cis*-monoenes [12]. Δ^9^ Stearoyl-ACP desaturases (SADs) efficiently desaturate C18:0 (stearic acid) to form *cis*-ω-9 C18:1 (Δ^9^ 18:1; oleic acid) and represent the archetypal AAD [13]. Some SADs also exhibit low levels of Δ^9^ palmitoyl-ACP desaturase (PAD) activity, thus forming *cis*-ω-7 C16:1 (Δ^9^ 16:1; palmitoleic acid) [14,15]. However, production of ω-7 monoenes in tissues storing oils enriched in ω-7 FAs relies on variant specialized PAD isoforms that display a distinct substrate specificity, with a preference for C16:0-ACP. Whereas SAD isoforms have been cloned and characterized in a relatively wide range of plant species, a limited number of PADs have been identified so far. A PAD-encoding cDNA was first cloned in cat’s claw vine [16]. More recently, two PAD isoforms named AAD2 and AAD3 were identified in *A*. *thaliana* [11,17]. Three-dimensional structures of these related, but functionally divergent, soluble desaturases have provided clues on how they recognize the chain length of substrates [18,19]. A hydrophobic channel within the enzymes accommodates FA substrates bound to ACP, and the side chains of amino acids lining the lower portion of this channel set constraints on the chain length of FA substrates. The structure of the channel of the archetype Δ^9^ SAD is highly conserved among plant species. This channel is deep enough to accommodate C18:0-ACP substrates. On the contrary, the bottom part of the substrate channel of Δ^9^ PADs is lined by hydrophobic residues with bulky lateral chains that reduce the depth of their substrate pockets and favor the binding of shorter C16:0-ACP substrates [17,19]. Despite similar enzymatic activities, Δ^9^ PADs from cat’s claw vine and *A*. *thaliana* do not have identical substrate channels, suggesting that these activities may have appeared independently during the evolution of AADs.

Due to the high economic value of plant ω-7s, there is an increasing interest in either finding new natural plant sources with potentially better agronomical performances or improving existing oilseed crops for better ω-7 production. Bryant et al. [11] determined the ω-7 content of endosperm oil in 10 inbred rapeseed varieties and reported significant variation, with the relative proportion of ω-7s reaching 47% in the endosperm of the Swede (Rutabaga) variety ‘Huguenot’. This enrichment in ω-7s concerns only the aleurone, where approximately 5% of the FA in rapeseed is stored. The authors suggested that extraction of ω-7s from such a variety could be economically viable provided that rapeseed is dehulled before oil extraction. Alternatively, approaches of metabolic engineering can be implemented for enhancing production of ω-7s in the main oil-storing compartment of oilseeds. This has been successfully achieved in *A*. *thaliana* and in crops such as camelina (*Camelina sativa*), soybean (*Glycine max*), and rapeseed [1,20,21]. These approaches all relied on a strong and seed-specific expression of PAD sequences either cloned from species naturally accumulating high ω-7 levels (cat’s claw vine) or obtained through the reengineering of archetypal SADs to achieve desired substrate specificity while retaining the stability and turnover characteristics of a paralog [22]. Additional metabolic modifications were then introduced in some of these systems to efficiently redirect fluxes for high levels of ω-7 accumulation in the host seed (reviewed in [23]). Examples include reducing the elongation and the export of C16:0 from the plastid to enhance substrate availability for the PADs. Ultimately, iterative optimization of their strategy led Nguyen et al. [1] to use an assembly of six transgenes yielding 60–65% ω-7 FAs in the oil of transgenic camelina seeds.

The evolutionary history of the AADs has led to the emergence of isoforms catalyzing the biosynthesis of ‘exotic’ monoenes such as the ω-7s, therefore allowing the specialization of oil metabolism in certain oleaginous species. Interestingly, recent studies have underlined that specialization of ω-7 metabolism also relied on the complex setup of dedicated transcriptional machinery able to precisely control the spatiotemporal expression of PAD genes. In *A*. *thaliana*, two closely related members of the MYB family of transcription factors (TFs), *MYB115* and *MYB118*, are transcriptionally induced at the onset of the maturation phase in the endosperm. In this compartment, they activate a set of common transcriptional targets such as the two PAD-coding genes *AAD2* and *AAD3*. Even though the molecular mechanism underlying this transcriptional activation has not been fully elucidated, the two MYBs were shown to be necessary for endosperm-specific activation of *AAD2* and *AAD3* and the subsequent accumulation of ω-7s in this seed compartment. Accordingly, the relative proportion of ω-7s is drastically reduced in the endosperm oil of *myb115 myb118* double mutants, just like in that of the *aad2 aad3* double mutants [17].

In the present report, we systematically examine the biotechnological potential of the actors of ω-7 metabolism newly isolated in *A*. *thaliana* for increasing ω-7 FA content in seeds. Our objective is to identify new gene sequences useful for metabolic engineering for efficient plant-based production of ω-7 FAs. We therefore placed coding sequences of *A*. *thaliana* PADs or their transcriptional activator MYB115 under the control of a promoter strongly induced in seeds and evaluated these different constructs in *A*. *thaliana*. Oils were obtained that exhibited ω-7 FA contents ranging from 10 to >50% of the total FAs. Even though all the constructs tested efficiently yielded expected compositional changes, direct overexpression of the *PADs* appeared more efficient than that of *MYB115* for stimulating ω-7 FA biosynthesis. Remarkably, these major compositional changes had no detrimental defect on seed germination.

## Materials and methods

### Plant material and growth conditions

Plants were cultured as described in Baud et al. [24]. To sample embryos and endosperm/seed coats, seeds were imbibed and dissected under an optical glass binocular magnifier with a scalpel and dissecting tweezers. Samples used for RNA extraction were frozen in liquid nitrogen immediately after dissection and then stored at -80°C. Weight determinations of seed samples were realized on a M2P balance (Sartorius, Aubagne, France). For germination assays, seeds were sown in triplicate in Petri dishes containing 0.5% (w/v) solidified agarose. After stratification, plates were kept in a growth cabinet (with continuous light at 25°C), and germination was scored based on radicle emergence.

### Constructs and plant transformation

The sequences of primers used for DNA amplification are indicated in S1 Table.

For construction of the *ProAT2S2*:*uidA*, *ProAT2S2*:*MYB115*, *ProAT2S2*:*AAD2*, and *ProAT2S2*:*AAD3* transgenes, cDNA was amplified with the proofreading Pfu Ultra DNA polymerase (Stratagene, Les Ulis, France) from the pGWB3 vector ([25]; *uidA*) or from a mixture of seed cDNA (Col-0 accession; *MYB115*, *AAD2*, and *AAD3*). The PCR products thus obtained were introduced by BP recombination into the pDONR207 entry vector (Invitrogen, Paris, France) and transferred into the Pro_AT2S2_-R1R2-HYGRO destination vector [26] by LR recombination. The resulting binary vectors were electroporated into the *Agrobacterium tumefaciens* C58C^1^ strain and used for agroinfiltration of flower buds of *A*. *thaliana* [27]. Primary transformants were selected on Murashige and Skoog medium containing hygromycin (50 mg.l^-1^). They were then transferred to soil for further characterization. The progeny of these primary transformants (T2 seeds) was subjected to segregation analyses, and lines segregating 3:1 for hygromycin resistance were selected (heterozygous lines, one insertion locus). T2 lines were then grown in a greenhouse, and their progeny (T3 seeds) was subjected to segregation analyses. Lines producing 100%-resistant plantlets were selected (homozygous lines, single insertion locus) and used for further analyses.

### RNA analyses

RNA extraction, reverse transcription, and real-time RT quantitative PCR were performed as previously described [28]. The sequences of primers used for real-time RT-qPCR are indicated in S2 Table. Purity of the different seed fractions sampled was assessed as described in Barthole et al. [29]. Briefly, marker genes for each of the seed fractions sampled, namely *ZHOUPI* (endosperm-specific) and *At2g23230* (embryo-specific), were quantified on cDNA prepared from these fractions, thus demonstrating that no significant contamination occurred between fractions.

### Fatty acid analyses

Gas chromatography analyses of total FAs were performed as previously described [10] on pools of *A*. *thaliana* seeds or seed fractions. The endosperm was analyzed with the seed coat attached as described in Penfield et al. [9]. The procedure did not bias our evaluation of the endosperm oil content since the seed coat does not accumulate storage lipids and undergo programmed cell death during seed maturation [10,30].

### Microscopy

Bright-field microscope observations and histochemical detection of GUS activity were carried out as previously described in Baud et al. [24].

### Accession numbers

Sequence data can be found in the GenBank/EMBL data libraries under the following accession numbers: *AAD2*, At4g02610; *AAD3*, At5g16230; *AT2S2*, At4g27140; *MYB115*, At5g40360; *MYB118*, At3g27785.

## Results

### Validation of a promoter sequence suitable for overexpression of genes of interest in zygotic tissues of the seed

**Fig 1:**
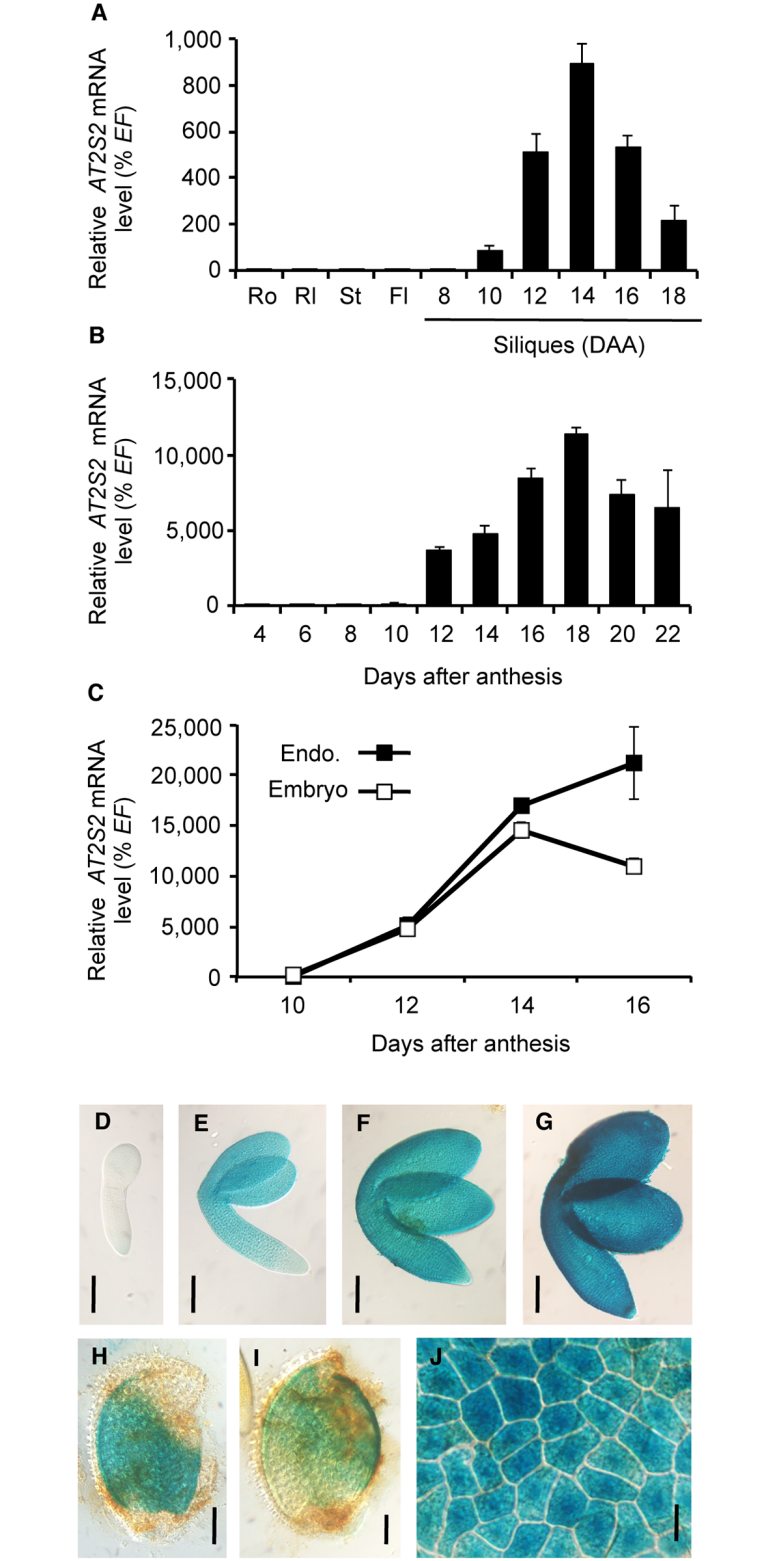
Validation of the *AT2S2* promoter sequence. (A-C) Analysis of relative mRNA accumulation of *AT2S2* was performed in different plant organs (A), in developing seeds (B), and in developmental series of endosperm (Endo.) and embryo fractions (C). The results obtained were standardized to the *EF1αA4* (*EF*) gene expression level. Values are the means and SE of three to six replicates carried out on cDNA dilutions obtained from three independent mRNA extractions. DAA, days after anthesis; Fl, flowers; Rl, rosette leaves; Ro, roots; St, stems. (D-J) Pattern of activity of the *ProAT2S2*:*uidA* cassette in maturing embryos harvested 10 (D), 12 (E), 14 (F), or 16 days after anthesis (DAA) (G) and in endosperm fractions harvested 14 (H) or 16 DAA (I). A close-up of a peeled endosperm layer aged 14 DAA is presented in (J). For histochemical detection of GUS activity, tissues were incubated 4 hours in a buffer containing 2 mM each of potassium ferrocyanide and potassium ferricyanide. Microscopy observations were performed using Nomarski optics. Bars = 100 μm in (D-I), 10 μm in (J).

Because of the importance of the transcriptional activation of PAD-coding genes by MYB115 and MYB118 for ω-7 production in *A*. *thaliana* seeds [17], these MYBs appeared as potential new targets for the engineering of seed oils enriched in ω-7 monoenes. Before evaluating the biotechnological potential of these TFs, we looked for a promoter sequence allowing their overexpression in the oil-storing compartments of seeds. The use of a *35S* promoter sequence for high and ubiquitous expression was not possible since the *Pro35Sdual*:*MYB115* and *Pro35Sdual*:*MYB118* cassettes dramatically affect plant growth and reproduction, yielding dwarfism and partial sterility [17,29]. To overcome the negative impact of a strong expression of *MYB115* or *MYB118* in non-seed tissues, we instead chose the promoter sequence of the *AT2S2* gene (At4g27150) coding for a seed storage protein. RNA gel blot analyses, *in situ* hybridization approaches, and laser-capture microdissection of maturing seeds followed by mRNA quantification using stringent analyses of Affymetrix ATH1 GeneChip hybridization data previously showed that *AT2S2* is highly induced in the endosperm and in the embryo of maturing seeds [31,32]. We verified this expression pattern by RT-qPCR on a set of cDNA prepared from a range of plant organs of the wild-type accession Columbia-0 (Col-0). To begin with, a range of plant organs were considered, and *AT2S2* appeared to be highly expressed in siliques (Fig 1A). To further characterize the expression pattern of *AT2S2*, a time-course analysis of *AT2S2* mRNA abundance was carried out in developing seeds excised from the siliques, which revealed a peak of transcript accumulation during maturation (Fig 1B). To gain further insight into the tissue specificity of *AT2S2* expression, maturing seeds were dissected. The two fractions thus obtained, embryo and endosperm/seed coat, were independently analyzed. A strong induction of *AT2S2* during the course of maturation was observed in the two seed fractions under study (Fig 1C). These data confirmed that the *AT2S2* promoter was well adapted for overexpressing genes of interest in maturing seeds.

A 984-bp *AT2S2* gene promoter fragment exhibiting a close association of RY and G-box elements essential for the activation of maturation genes by the master regulators of seed maturation [33] was then cloned (S3 Table) and transcriptionally fused to the *uidA* reporter gene for validating its activity. The corresponding construct was assayed for the resulting *uidA* expression pattern in transgenic *A*. *thaliana* lines. Intense GUS activity was observed in the embryos (Fig 1D–1G) and in the endosperm of maturing seeds (Fig 1H–1J). This promoter fragment was therefore used for driving the expression of *MYB115* and *MYB118* in seeds.

### Overexpression of *MYB115* in seeds

Both *ProAT2S2*:*MYB115* and *ProAT2S2*:*MYB118* cassettes were prepared (see Methods). Unfortunately, we failed to recover *ProAT2S2*:*MYB118* transformants. The corresponding antibiotic-resistant plantlets selected *in vitro* did not survive when transferred to soil. On the contrary, transgenic lines carrying the *ProAT2S2*:*MYB115* construct could be generated. Five independent transformants with a single insertion locus were characterized. Homozygous lines were grown under controlled conditions, and neither vegetative growth nor fertility was affected in these lines, despite moderate activity of the *ProAT2S2* promoter used in vegetative and in reproductive organs (S1 Fig). Likewise, whole-mount clearing of developing seeds showed that the structure and early development of the three tissues composing the seeds were unmodified in a *ProAT2S2*:*MYB115* background (S2 Fig).

**Fig 2:**
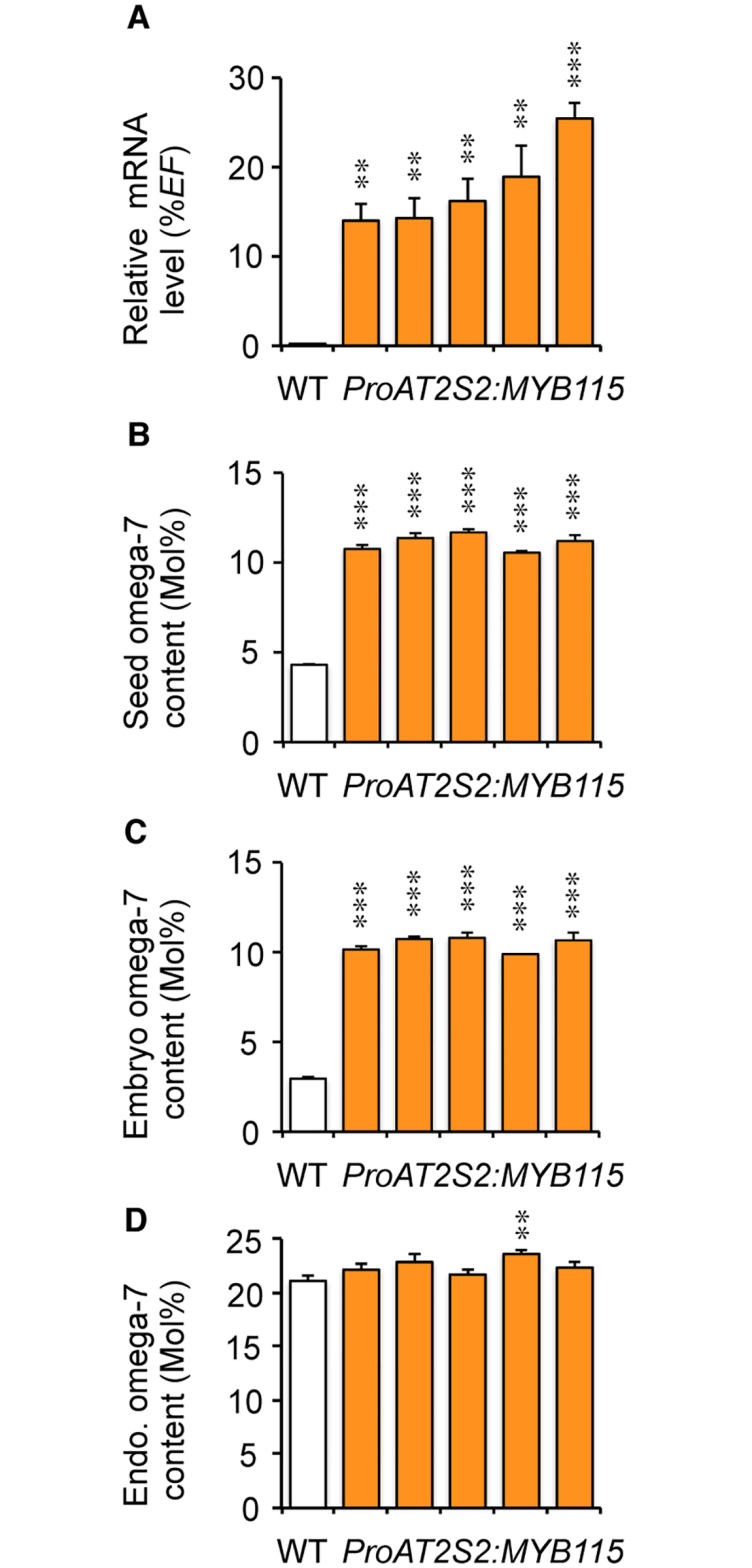
Increased omega-7 fatty acid content of *ProAT2S2*:*MYB115* seeds. The five independent transformants considered, TCR4, TAR3, TXR2, TGR5, and TJR2 (in order from left to right), are displayed in the same order in the four graphs in the figure. (A) RT-qPCR analysis of transcript abundance in cDNA prepared from maturing seeds aged 14 DAA to assess efficient overexpression of the transgene. Values are the means and SE of three to 12 replicates performed on cDNA dilutions obtained from three independent mRNA extractions. (B-D) Relative proportion of ω-7 fatty acids (*cis*-ω-7 C16:1, *cis*-ω-7 C18:1, and *cis*-ω-7 C20:1) in mature dry seeds (B), in embryos (C), and in endosperm fractions (D) dissected from mature seeds. Values are the means and SE of five replicates performed on batches of 20 seeds from five plants. Asterisks indicate significant differences from the wild type according to *t*-test at *** P<0.001 and **P<0.01, respectively.

Using a RT-qPCR strategy, *MYB115* mRNA level was quantified in maturing seeds of the transgenic lines (Fig 2A). Expression levels ranging from 14% to 25% of the *EF1αA4* (At5g60390) mRNA level (constitutive expression; [34]) were detected 14 days after anthesis (DAA), indicating that *MYB115* was efficiently overexpressed in these lines (from 660- to 1,170-fold compared to expression in the wild type).

**Fig 3:**
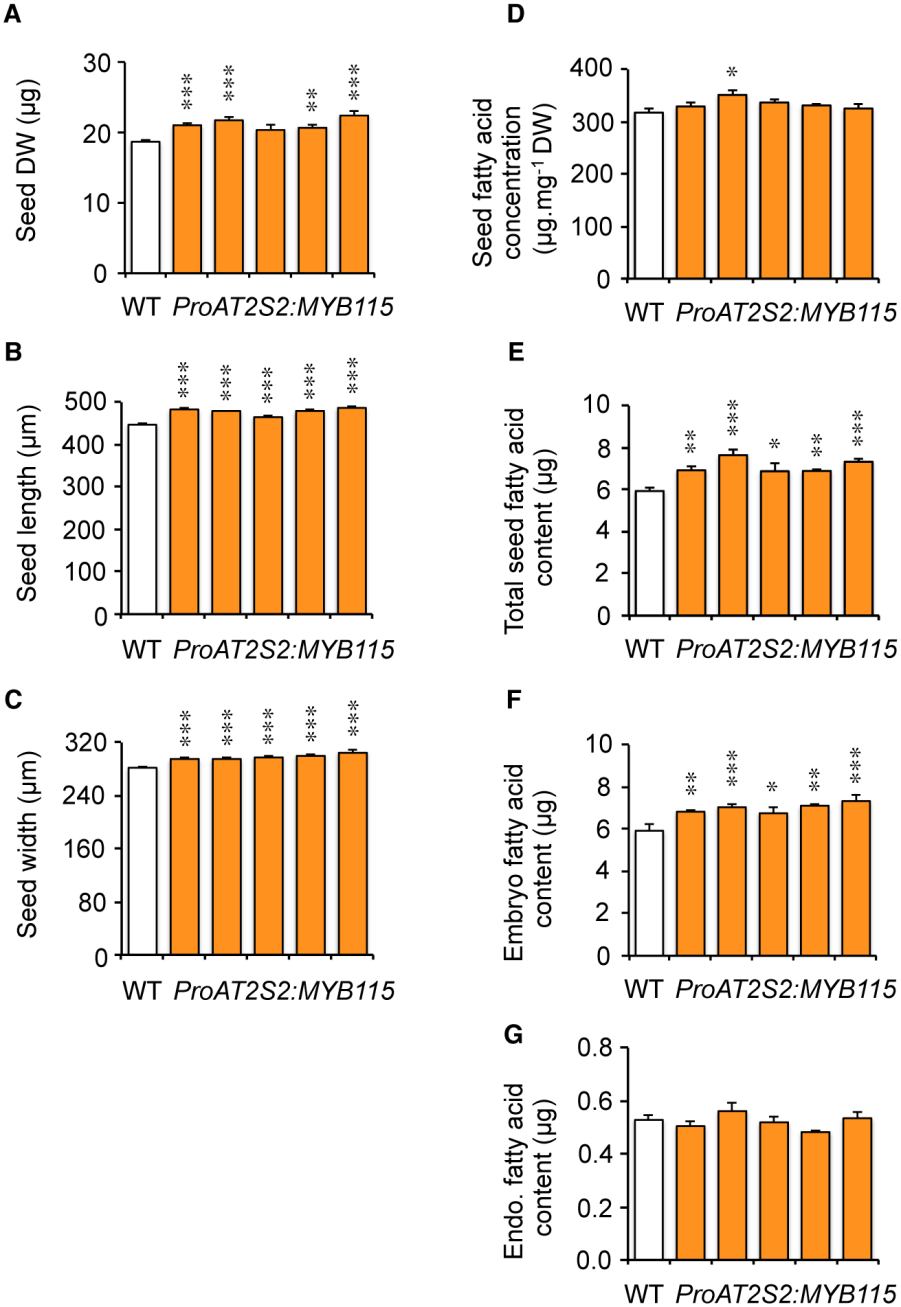
Characterization of transgenic seeds overexpressing *MYB115*. The five independent transformants considered, TCR4, TAR3, TXR2, TGR5, and TJR2 (in order from left to right), are displayed in the same order in every graph in the figure. (A) Mature seed dry weight. Values are the means and SE of five replicates carried out on batches of 20 seeds from five plants. (B) Mature seed length. Values are the means and SE of 100 measurements carried out on seeds from five plants. (C) Mature seed width. Values are the means and SE of 100 measurements carried out on seeds from five plants. (D, E) Total fatty acid content of mature dry seeds, expressed in μg.mg^-1^ DW (D) or in μg per seed (E). Values are the means and SE of five replicates carried out on batches of 20 seeds from five plants. (F) Total seed fatty acid content of embryos dissected from mature dry seeds. Values are the means and SE of five replicates carried out on batches of 20 embryos from five plants. (G) Total seed fatty acid content of endosperm fractions dissected from mature dry seeds. Values are the means and SE of five replicates carried out on batches of 20 endosperm fractions from five plants. Asterisks indicate significant differences from the wild type according to *t*-test at *** P<0.001, **P<0.01, and *P<0.05, respectively.

To evaluate the effect of *MYB115* overexpression on seed filling, whole mature dry seeds were first analyzed. Seed dry weight and total FA content were significantly increased in the transgenic lines (from 10% to 20%) as a consequence of increased seed dimensions (Fig 3A–3E). However, FA concentration in these seeds remained unchanged. Mature seeds were then dissected. The two fractions obtained, embryo and endosperm/seed coat, were collected separately before total FA quantification by gas chromatography. A significant increase in total FAs was measured in transgenic embryos (Fig 3F). On the contrary, the total amount of FAs stored in the endosperm fraction was unmodified (Fig 3G). The FA composition of the oil stored in transgenic seeds was then examined, with a particular focus placed on monoenes of the ω-7 series. At the whole seed level, overexpression of *MYB115* yielded a strong enrichment in ω-7 FAs (*cis*-ω-7 C16:1, *cis*-ω-7 C18:1, and *cis*-ω-7 C20:1), the relative proportion of which was more than doubled with respect to the wild type (Fig 2B; S4 Table). This phenotype hid contrasting results in the two zygotic compartments of the seed, with the concentration of ω-7 being unchanged in endosperm oil, whereas that of embryo oil was more than tripled (Fig 2C and 2D; S5 and S6 Tables).

**Fig 4:**
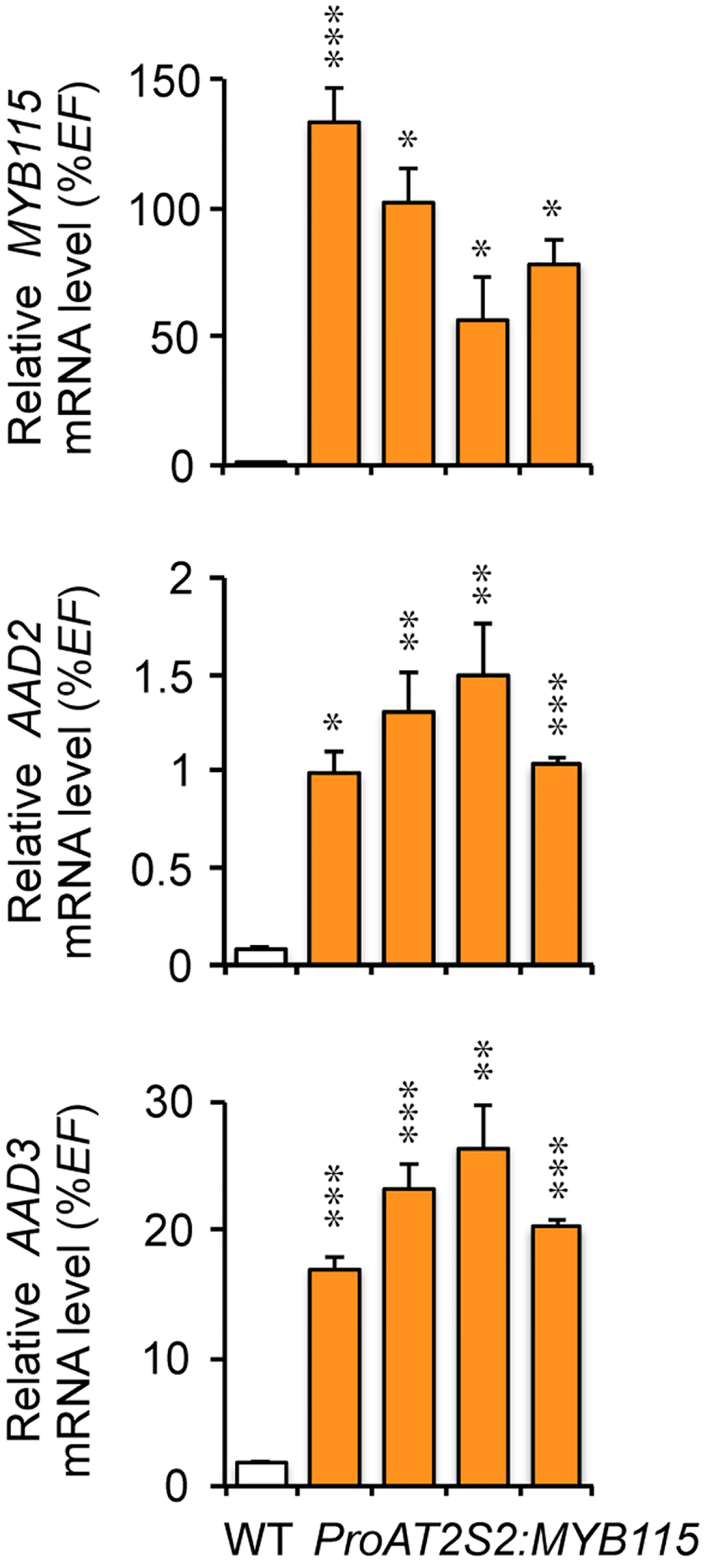
Analysis of *MYB115*, *AAD2*, and *AAD3* transcript levels by quantitative RT-PCR in *ProAT2S2*:*MYB115* embryos. The four independent transformants considered, TAR3, TXR2, TGR5, and TJR2 (in order from left to right), are displayed in the same order in the three graphs in the figure. RT-qPCR analysis of transcript abundance in cDNA prepared from excised embryos aged 14 DAA was carried out to assess efficient overexpression of the transgene (*MYB115*) and of its targets (*AAD2* and *AAD3*). Values are the means and SE of three replicates performed on cDNA dilutions obtained from three independent mRNA extractions. Asterisks indicate significant differences from the wild type according to *t*-test at *** P<0.001, **P<0.01, and *P<0.05, respectively.

To test whether the increase in ω-7 content measured in transgenic seeds correlated with higher *AAD2* and *AAD3* transcript abundance, we first quantified the relative accumulation of these transcripts on cDNA prepared from whole seeds harvested 14 DAA. There was no significant variation of the *AAD* transcript levels in transgenic seeds with respect to the wild type (S3 Fig). However, a tendency for increased *AAD3* mRNA accumulation in transgenic seeds was observed. FA analyses carried out on dissected seeds (see above) led us to hypothesize that statistically significant variation of the *AAD* transcript levels occurring in transgenic embryos might be hidden by the naturally elevated but unmodified abundance of these transcripts in corresponding albumens. For a subset of lines, we therefore prepared cDNA from dissected embryos and verified that increased embryo ω-7 levels correlated with enhanced transcriptional activation of both *AAD2* and *AAD3* in this seed compartment (Fig 4). Unfortunately, the very small size of the endosperm fraction prevented us from collecting sufficient material on the different lines considered to perform similar analyses on this fraction. As a consequence, we ignore whether the *AADs* were efficiently overexpressed in the albumen of transgenic seeds.

### Overexpression of *AAD2* or *AAD3* in seeds

**Fig 5:**
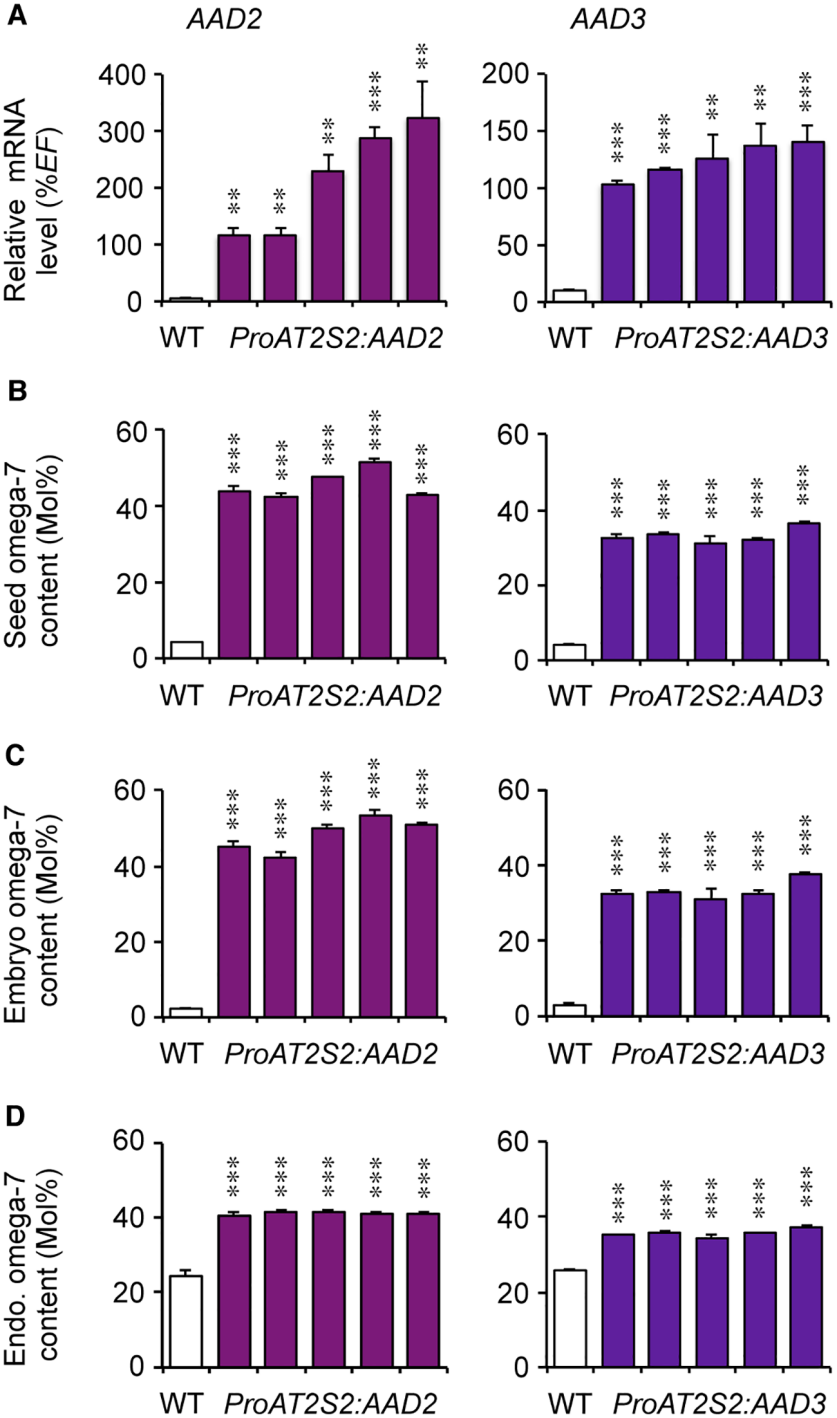
Increased ω-7 fatty acid content in seeds of transgenic *A*. *thaliana* lines overexpressing *AAD2* or *AAD3*. Five independent lines are presented for each construct tested. The five *ProAT2S2*:*AAD2* transformants considered, T2R3, T19R1, T7R3, T17R1, and T13R8 (in order from left to right); and the five *ProAT2S2*:*AAD3* transformants considered, T7R1, T19R5, T16R1, T20R5, and T13R2 (in order from left to right), are always displayed in the same order in the graphs in the figure. (A) RT-qPCR analysis of transcript abundance in cDNA prepared from maturing seeds aged 14 DAA to assess efficient overexpression of the transgenes (noted in italics). Values are the means and SE of three to six replicates performed on cDNA dilutions obtained from three independent mRNA extractions. (B-D) Relative proportion of ω-7 fatty acids (*cis*-ω-7 C16:1, *cis*-ω-7 C18:1, and *cis*-ω-7 C20:1) in mature dry seeds (B), in embryos (C), and in endosperm fractions (D) dissected from mature seeds. Values are the means and SE of five replicates performed on batches of 20 seeds from five plants. Asterisks indicate significant differences from the wild type according to *t*-test at *** P<0.001 and **P<0.01, respectively.

To compare the effect of *MYB115* overexpression on ω-7 accumulation with that of one or the other of the *PADs* induced by MYB115, transgenic plants carrying either the *ProAT2S2*:*AAD2* construct or the *ProAT2S2*:*AAD3* construct were then obtained. For each construct, five independent transformants were considered. With a RT-qPCR strategy, *AAD* mRNA levels were quantified in maturing seeds 14 DAA (Fig 5A). Significantly increased *AAD2* mRNA levels were detected in maturing *ProAT2S2*:*AAD2* seeds, indicating that the transgene was efficiently overexpressed (from 20- to 60-fold compared to the wild type). Similarly, a significant overexpression of *AAD3* (from 10- to 14-fold compared to the wild type) was measured in the *ProAT2S2*:*AAD3* lines. To evaluate the effect of *AAD2* and *AAD3* overexpression on seed filling, whole mature dry seeds were then subjected to fine biochemical analysis. Dry weight of the transgenic mature seeds was unmodified (S4A Fig). Likewise, the overall oil content of the two zygotic compartments of the seeds was unchanged. The FA composition of the oil stored in transgenic seeds was then examined. At the seed level, overexpression of *AAD2* and *AAD3* yielded a strong enrichment in ω-7s, the relative proportion of which was multiplied by eight (*AAD3*) to 10 (*AAD2*) with respect to the wild type (Fig 5B; S4 Table). The ω-7 FA contents measured at the seed level faithfully reflected the relative proportions of ω-7 FA species observed both in the embryo and in the endosperm fractions of transgenic seeds (Fig 5C and 5D; S5 and S6 Tables).

### Increased seed omega-7 content does not affect germination

**Fig 6:**
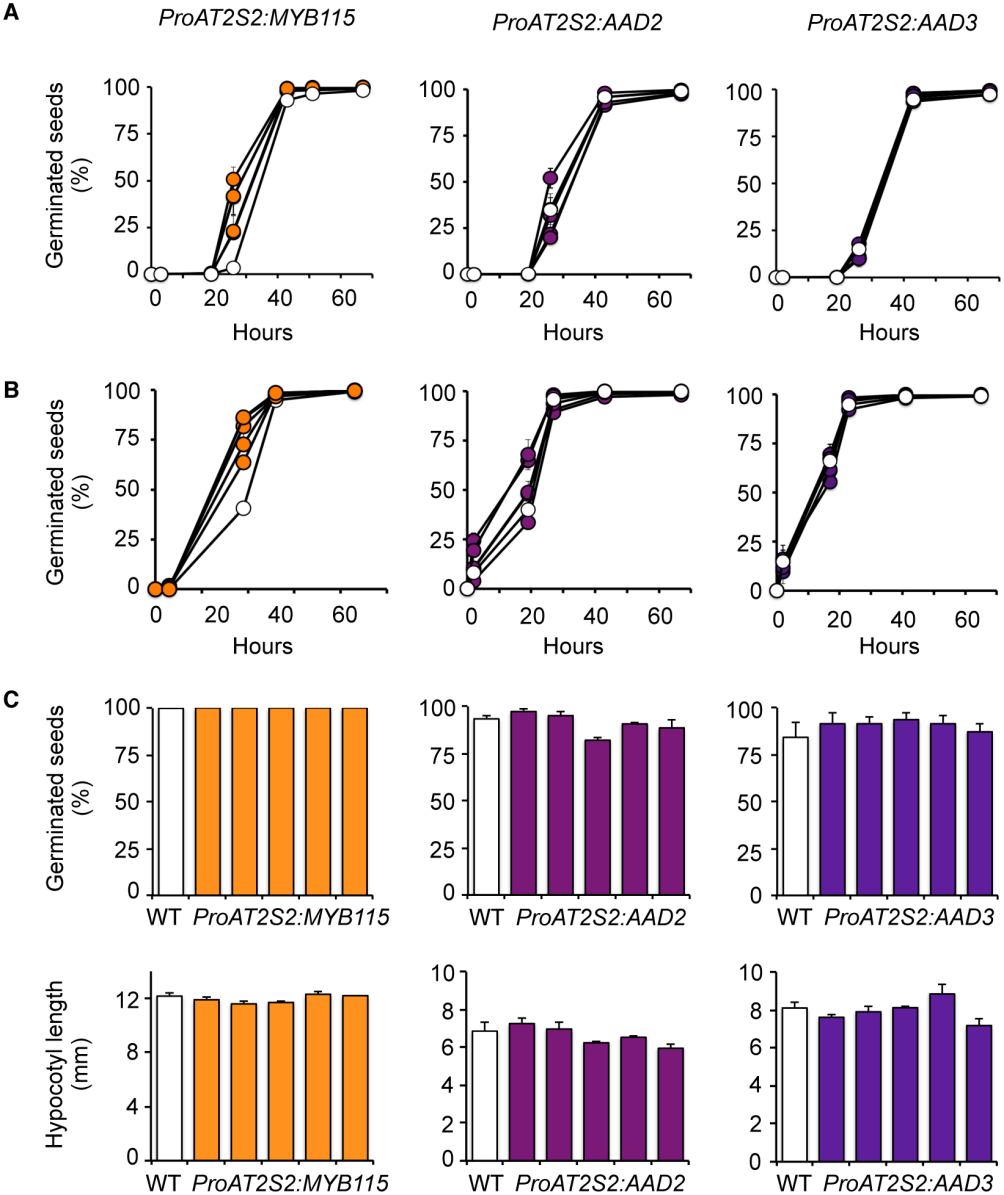
Effect of increased omega-7 content on seed germination. Five independent transgenic lines are presented for each construct tested. (A) Germination time-courses of unstratified seeds. White circles represent wild-type seeds, and filled circles represent transgenic seeds. (B) Germination time-courses of seeds after 5 days of cold stratification at 4°C in the dark. White circles represent wild-type seeds, and filled circles represent transgenic seeds. (C) Germination rates of seeds cold-stratified 5 days at 4°C, then exposed to a 1-hour light impulse, and finally germinated in the dark at 25°C for 7 days. The five independent *ProAT2S2*:*MYB115* lines considered, TAR3, TCR4, TGR5, TJR2, and TXR2 (in order from left to right); the five independent *ProAT2S2*:*AAD2* transformants considered, T2R3, T19R1, T7R3, T17R1, and T13R8 (in order from left to right); and the five independent *ProAT2S2*:*AAD3* transformants considered, T7R1, T19R5, T16R1, T20R5, and T13R2 (in order from left to right), are always displayed in the same order in the graphs in the figure.

Since drastic alterations of FA composition of seed oils sometimes affect triacylglycerol lipolysis during germination and seed vigor [35], germination of the engineered seeds was then examined (Fig 6). Under standard light conditions, *ProAT2S2*:*MYB115* seeds exhibited a transient but reproducible increase of the germination rate 24 hours after the first exposure to the light. This phenotype was apparently not linked with the increased ω-7 content of these seeds, given that the germination rate of seeds overexpressing *AAD2* or *AAD3* was similar to that of wild-type seeds. We then indirectly evaluated the ability of transgenic seedlings to remobilize triacylglycerols enriched in ω-7 FAs. For this purpose, cold-stratified seeds were submitted to a brief light impulse aimed at triggering the germination process and then placed in the dark, where carbon fixation by photosynthesis is prevented. After one week, the germination rate of engineered seeds was similar to that of wild-type seeds. The hypocotyl length of the transgenic etiolated seedlings thus obtained was unmodified, suggesting that triacylglycerols enriched in ω-7 FAs were efficiently remobilized.

## Discussion

Iterative optimization of unusual FA production in seeds not only requires the stacking of multiple traits [36], but also implies the evaluation and comparison of different gene versions for a given trait and that of regulatory elements for driving the expression of selected transgenes. The results presented in this study demonstrate that overexpression of either of the two *A*. *thaliana* PADs in seeds is sufficient to partially shift monoene production from ω-9 to ω-7 FAs. This result can be attained both by overexpression of the desaturase-coding sequences placed under the control of the *AT2S2* promoter and by overexpression of one of their transcriptional activators, such as MYB115. With regard to the efficiency of the different strategies evaluated, the latter appears to have lower efficiency. The relative ω-7 FA content of *ProAT2S2*:*MYB115* seeds is five time less than that of *ProAT2S2*:*AAD2* seeds on average. Further, increased ω-7 levels are observed only in embryos of *MYB115* overexpressing lines when the production of ω-7 monoenes is increased both in the embryo and in the endosperm of *ProAT2S2*:*AAD2* and *ProAT2S2*:*AAD3* seeds. These results suggest that transcriptional activation of PAD genes constitutes the main limiting factor in the control of ω-7 accumulation in *A*. *thaliana* seeds. This is in agreement with the QTL approach described by Bryant et al. [11], which led to the identification of a single major QTL determining the ω-7 FA content in seeds, the peak of which colocalized with *AAD3*. Other actors involved in the biosynthesis (acyl-carrier proteins and ferredoxins), the export, or the acylation of ω-7 monoenes seem to be expressed in seeds at a level sufficient to support an important shift from ω-9 to ω-7 FA production. It is tempting to speculate that the substrate range of the enzymes of lipid metabolism using ω-9 monoenes in a wild-type context is broad enough to efficiently metabolize ω-7 monoenes in a transgenic background. This is probably due to the fact that the structures of ω-9 and ω-7 FAs are similar enough. The structure of *cis*-ω-12 C18:1 (petroselenic acid) or *cis*-ω-10 C16:1 (sapienic acid) is more divergent. These monoenes are accumulated at high levels in seeds of *Coriandrum sativum* and *Thunbergia alata*, respectively. Specialized AADs catalyzing the synthesis of these unusual monoenes have been identified and characterized [37,38]. Expression of the corresponding cDNAs in seeds of *A*. *thaliana* yielded limited accumulation (<15 mol%) of the corresponding FAs [39], suggesting that specialized actors other than the AADs [40–42] may be required for efficient channeling of these unusual monoene species.

The contrasted seed ω-7 contents measured in lines overexpressing *AAD2* and *AAD3* (Fig 5; Table 1) demonstrate that *AAD2* constitutes a better candidate for ω-7 biosynthesis in an *A*. *thaliana* context. A first reason for this may reside in the improved stability of *AAD2* mRNAs, as denoted by the higher transcript levels measured in the seeds of several *ProAT2S2*:*AAD2* lines (Fig 5). Even in comparisons of *ProAT2S2*:*AAD2* and *ProAT2S2*:*AAD3* lines exhibiting roughly equivalent levels of transgene expression, the lines overexpressing *AAD2* always yield the highest seed ω-7 contents, suggesting that post-translational mechanisms also contribute to favor the activity of AAD2 over that of AAD3. Distinct characteristics of the two desaturases, such as protein stability, kinetic properties, or ability to interact with protein partners may account for these differences. In a wild-type context, however, the contribution of AAD3 to seed ω-7 FA biosynthesis is more important than that of AAD2, as indicated by the phenotypes of the corresponding single mutants [17] and the results of the QTL approach carried out by Bryant et al. [11]. The contrasted expression levels of the two desaturases in a wild-type context help explain these apparent discrepancies: in maturing seeds of the wild type, *AAD3* mRNAs accumulate at significantly higher levels, suggesting that the activity of the *AAD3* promoter is higher than that of the *AAD2* promoter [15,17]. A functional analysis of the two promoters will be required to elucidate the molecular bases of their contrasted activities.

**Table 1:** Comparison of the omega-7 fatty acid content of seeds from engineered lines of *A*. *thaliana* obtained by different groups.

Genotype	Fatty acid composition (mol% of total fatty acids)				Reference
	C16:1 omega-7	C18:1 omega-7	C20:1 omega-7	Total omega-7	
*ProAT2S2*:*AAD2* (Col-0)	4.26	30.21	17.03	51.50	This study
*ProAT2S2*:*AAD3* (Col-0)	3.18	20.76	12.31	36.45	This study
*ProNAP*:*DuPAD* (WS)	2.38	14.02	11.50	27.90	[20]
*ProPHAS*:*COM25*	1.6	12.8	n.d.	n.d.	[21]

Bondaruk et al. [20] used a PAD cDNA from cat’s claw (*Doxantha unguis-cati* L.) cloned downstream of a *NAPIN* promoter (from *Brassica napus*). Nguyen et al. [21] used a variant of the castor (*Ricinus communis*) Δ^9^-stearoyl-ACP desaturase named Com25 and obtained from enzyme evolution experiments to enhance the Δ^9^-palmitoyl-ACP desaturase activity of the enzyme. The corresponding cDNA was cloned under the control of a *PHASEOLIN* promoter (from *Phaseolus vulgaris*). The fatty acid composition of the best lines characterized (when available) was reported in this table. The accession of the engineered lines is indicated, when known, between brackets. n.d., not determined.

Comparing strategies of metabolic engineering implemented by different groups to accumulate ω-7 monoenes in *A*. *thaliana* seeds by means of the overexpression of PAD-coding sequences originating from different organisms is rendered difficult by the different promoters and accessions used (Table 1). Despite the fact that caution should be exercised with this comparison, the *ProAT2S2*:*AAD2* construct assessed in this study appears unambiguously to be a particularly efficient tool to stimulate ω-7 biosynthesis in seeds of *A*. *thaliana*. It would now be interesting to test the efficiency of this construct in other Brassicaceae species, such as camelina, before making use of it in a final assembly of several transgenes for further improvement of the seed ω-7 content. Nguyen and colleagues [1,21] indeed established that additional increases in ω-7 monoene production could be achieved by appropriate redirection of metabolic flux in maturing seeds.

The *ProAT2S2*:*MYB115* construct, though less efficient for promoting ω-7 FA biosynthesis in *A*. *thaliana* seeds, contributes to improve other seed traits of agronomic importance. The size of the *ProAT2S2*:*MYB115* seeds is first increased by 10–20% on average without any reduction of the total FA concentration so that the total lipid content of mature dry seeds is also raised by 10–20% (Fig 3). When examining the germination of the *ProAT2S2*:*MYB115* seeds, a transient but significantly improved rate of germination is then observed 24 hours after the first exposure to light (Fig 6). These phenotypes are independent of the seed ω-7 content since they are not observed in seeds of the *ProAT2S2*:*AAD2* and *ProAT2S2*:*AAD3* lines. Target genes of MYB115 other than *AAD2* and *AAD3* may contribute to control these important seed traits. *In vivo* chromatin immunoprecipitation experiments would be useful to characterize the spectrum of putative targets of MYB115 and identify candidate genes that may take part in the control of seed development and physiology. Since MYB118 also participates in the transcriptional activation of PAD-coding genes in *A*. *thaliana* seeds, this transcription factor constituted a promising candidate for stimulating ω-7 production by biotechnological means. Unfortunately, transgenic *ProAT2S2*:*MYB118* plantlets selected *in vitro* did not survive when transferred to soil. This suggests that the *in planta* functions of the closely related MYB115 and MYB118 TFs are not completely overlapping, even though they share several transcriptional targets.

In summary, we assessed the efficiency of new molecular tools for metabolically engineering the production of ω-7 monoenes in plants, and we showed that high ω-7 levels (>50%) could be attained in seeds of *A*. *thaliana* by overexpressing an endogenous PAD isoform, AAD2. The corresponding construct may now be tested, alone or in combination with others, in oleaginous species of agronomic importance. More importantly, this research invites us to further characterize and exploit, when available, the genetic resources (gene versions and promoter sequences) originating from the species to be transformed. If numerous reports have put forward the advantages of the introgression of exogenous genetic material (e.g. limitation of gene silencing and of negative post-translational regulations of enzymes), it seems that in the particular case of ω-7 FA biosynthesis, further research might benefit by taking advantage of endogenous genetic resources. Therefore, this study may set the foundation for implementing new strategies to develop plant platforms dedicated to the production of ω-7 monoenes.

## Supporting information

S1 Fig Complementary results for the characterization of *AT2S2* promoter activity. Pattern of activity of the *ProAT2S2*:*uidA* cassette in rosette (A) and cauline leaves (B), in inflorescences (C), and in flowers (D). For histochemical detection of GUS activity, tissues were incubated overnight in a buffer containing 2 mM each of potassium ferrocyanide and potassium ferricyanide. Microscopy observations were performed using Nomarski optics. Bars = 5 mm in (A-C), 500 μm in (D).
(TIF)

S2 Fig Characterization of early seed development in *ProAT2S2*:*MYB115* lines. Five independent *ProAT2S2*:*MYB115* lines are presented: TAR3, TCR4, TGR5, TJR2, and TXR2. (A) Observation of seed development. Whole mounts of early developing seeds (from 4 to 8 DAA) and of maturing embryos (10 and 12 DAA) were observed with Nomarski optics. Bars = 50 μm. (B) Observation of maturing endosperm. Whole mounts of peeled endosperms (14 DAA) were observed with Nomarksi optics. Cross-sections (upper panel) and lateral views (lower panel) are presented. Bars = 20 μm. DAA, days after anthesis; WT, wild type.
(TIF)

S3 Fig Analysis of *AAD2* and *AAD3* transcript levels by quantitative RT-PCR in *ProAT2S2*:*MYB115* seeds. The five independent transformants considered are TCR4, TAR3, TXR2, TGR5, and TJR2 (in order from left to right). RT-qPCR analysis of transcript abundance in cDNA prepared from excised embryos aged 14 DAA was carried out to assess efficient overexpression of *AAD2* and *AAD3*. Values are the means and SE of six replicates performed on cDNA dilutions obtained from three independent mRNA extractions.
(TIF)

S4 Fig Complementary results for the characterization of *A*. *thaliana* lines overexpressing *AAD2* or *AAD3*. The five independent *ProAT2S2*:*AAD2* transformants considered, T2R3, T19R1, T7R3, T17R1, and T13R8 (in order from left to right) and the five independent *ProAT2S2*:*AAD3* transformants considered, T7R1, T19R5, T16R1, T20R5, and T13R2 (in order from left to right), are always displayed in the same order in the graphs in the figure. (A) Mature seed dry weight. (B) Total fatty acid content of mature dry seeds, expressed in μg.mg^-1^ DW. (C) Total seed fatty acid content of embryos dissected from mature dry seeds. (D) Total seed fatty acid content of endosperm fractions dissected from mature dry seeds. Values are the means and SE of five replicates carried out on batches of 20 individuals from five plants. Asterisks indicate significant differences from the wild type according to *t*-test at **P<0.01 and *P<0.05, respectively.
(TIF)

S1 Table Primers used for construct preparation. (PDF)

S2 Table Primers used for quantitative RT-PCR. (PDF)

S3 Table At4g27140/*AT2S2* promoter sequence (5’→3’). (PDF)

S4 Table Total fatty acid composition (in mol%) of seeds from engineered lines of *A*. *thaliana*. (PDF)

S5 Table Total fatty acid composition (in mol%) of endosperm fractions dissected from seeds of engineered lines of *A*. *thaliana*. (PDF)

S6 Table Total fatty acid composition (in Mol%) of embryos dissected from seeds of engineered lines of Arabidopsis. (PDF)
