# Supplementary figures and images for: Overexpression of MYB115, AAD2, or AAD3 in Arabidopsis thaliana seeds yields contrasting omega-7 contents
Source: PLoS One. 2018 Jan 30;13(1):e0192156. doi: 10.1371/journal.pone.0192156 (PMC5790276; doi:10.1371/journal.pone.0192156)

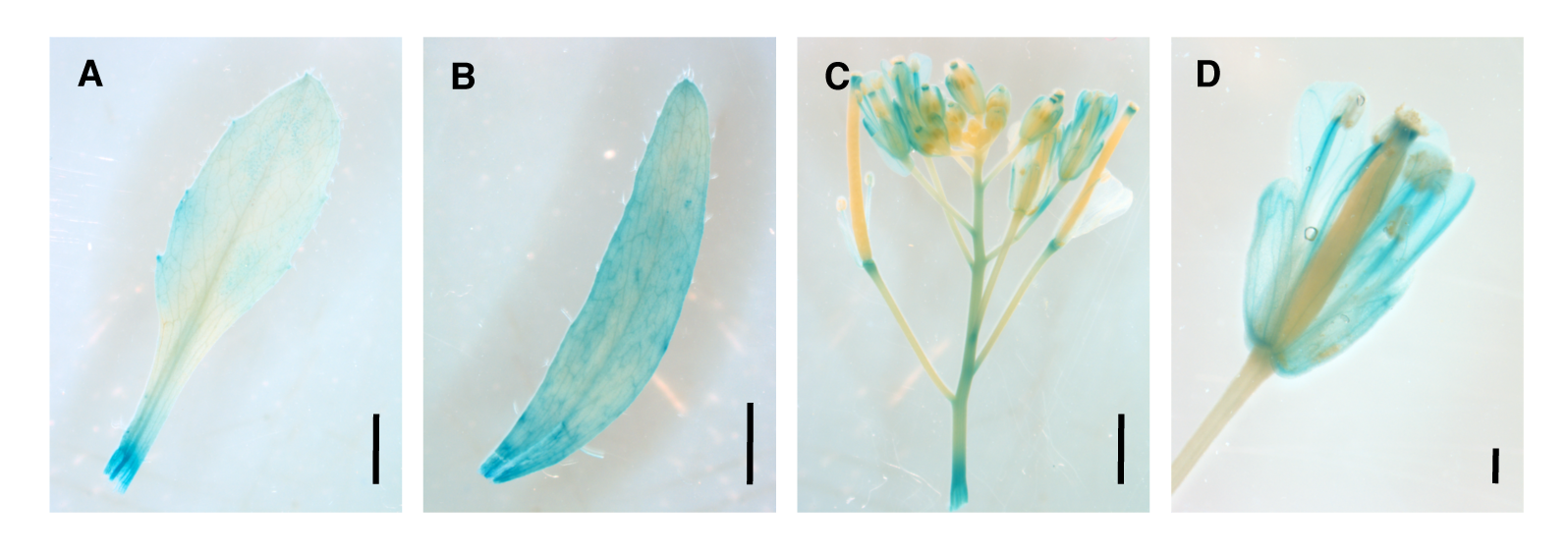

Supplement: S1 Fig — Pattern of activity of the ProAT2S2:uidA cassette in rosette (A) and cauline leaves (B), in inflorescences (C), and in flowers (D). For histochemical detection of GUS activity, tissues were incubated overnight in a buffer containing 2 mM each of potassium ferrocyanide and potassium ferricyanide. Microscopy observations were performed using Nomarski optics. Bars = 5 mm in (A-C), 500 μm in (D). (TIF) [file pone.0192156.s001.tif]

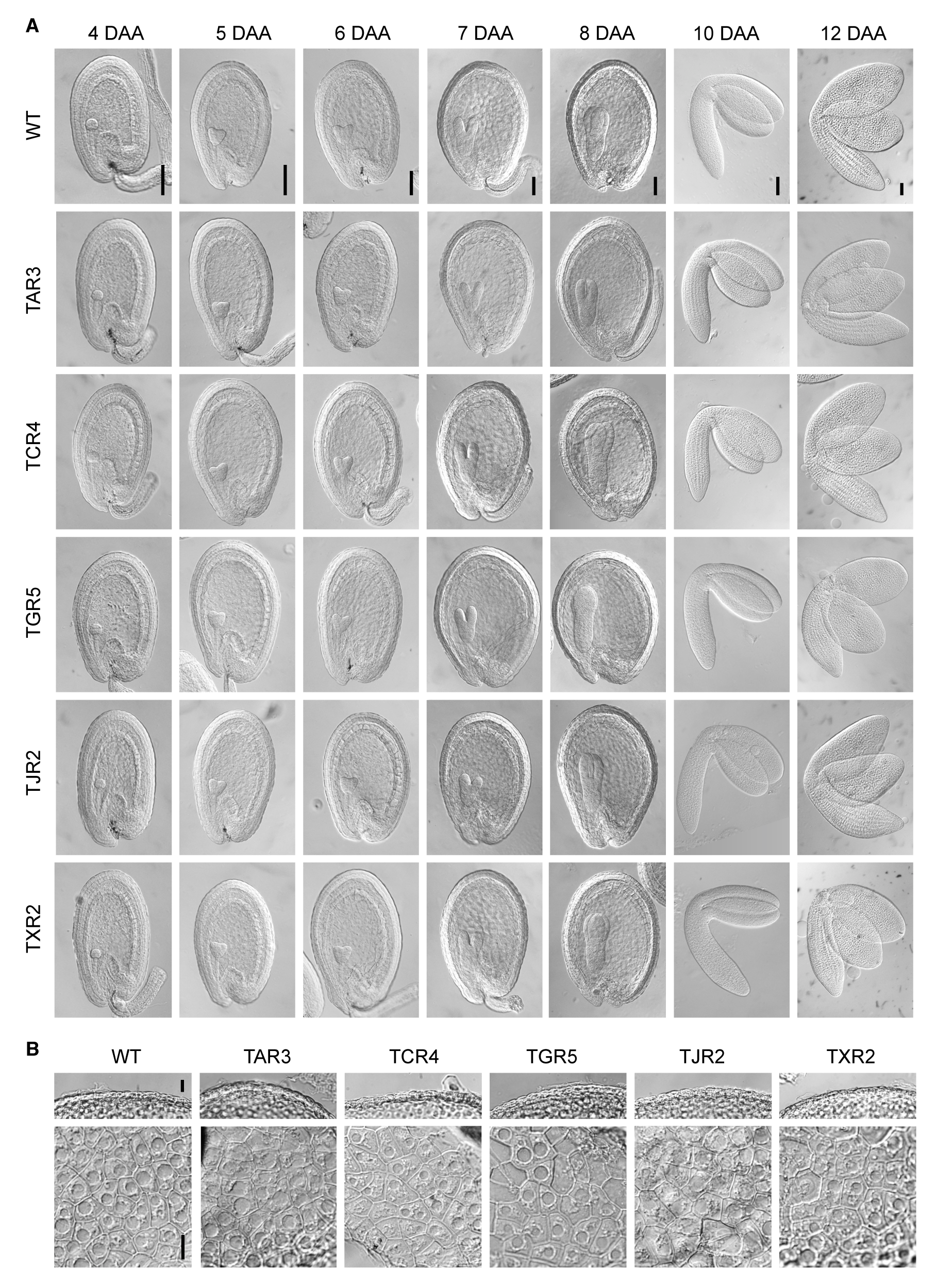

Supplement: S2 Fig — Five independent ProAT2S2:MYB115 lines are presented: TAR3, TCR4, TGR5, TJR2, and TXR2. (A) Observation of seed development. Whole mounts of early developing seeds (from 4 to 8 DAA) and of maturing embryos (10 and 12 DAA) were observed with Nomarski optics. Bars = 50 μm. (B) Observation of maturing endosperm. Whole mounts of peeled endosperms (14 DAA) were observed with Nomarksi optics. Cross-sections (upper panel) and lateral views (lower panel) are presented. Bars = 20 μm. DAA, days after anthesis; WT, wild type. (TIF) [file pone.0192156.s002.tif]

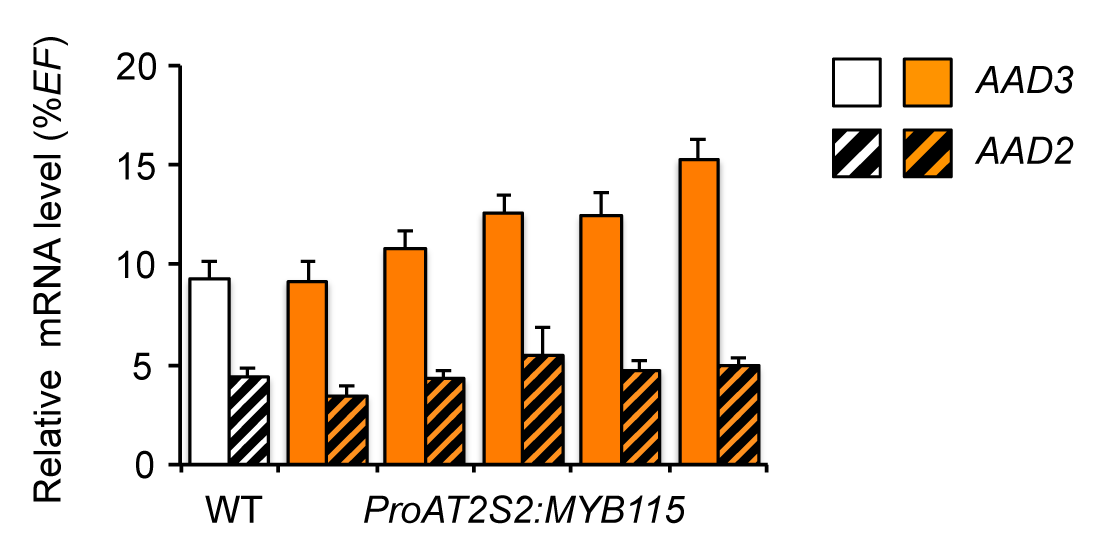

Supplement: S3 Fig — The five independent transformants considered are TCR4, TAR3, TXR2, TGR5, and TJR2 (in order from left to right). RT-qPCR analysis of transcript abundance in cDNA prepared from excised embryos aged 14 DAA was carried out to assess efficient overexpression of AAD2 and AAD3. Values are the means and SE of six replicates performed on cDNA dilutions obtained from three independent mRNA extractions. (TIF) [file pone.0192156.s003.tif]

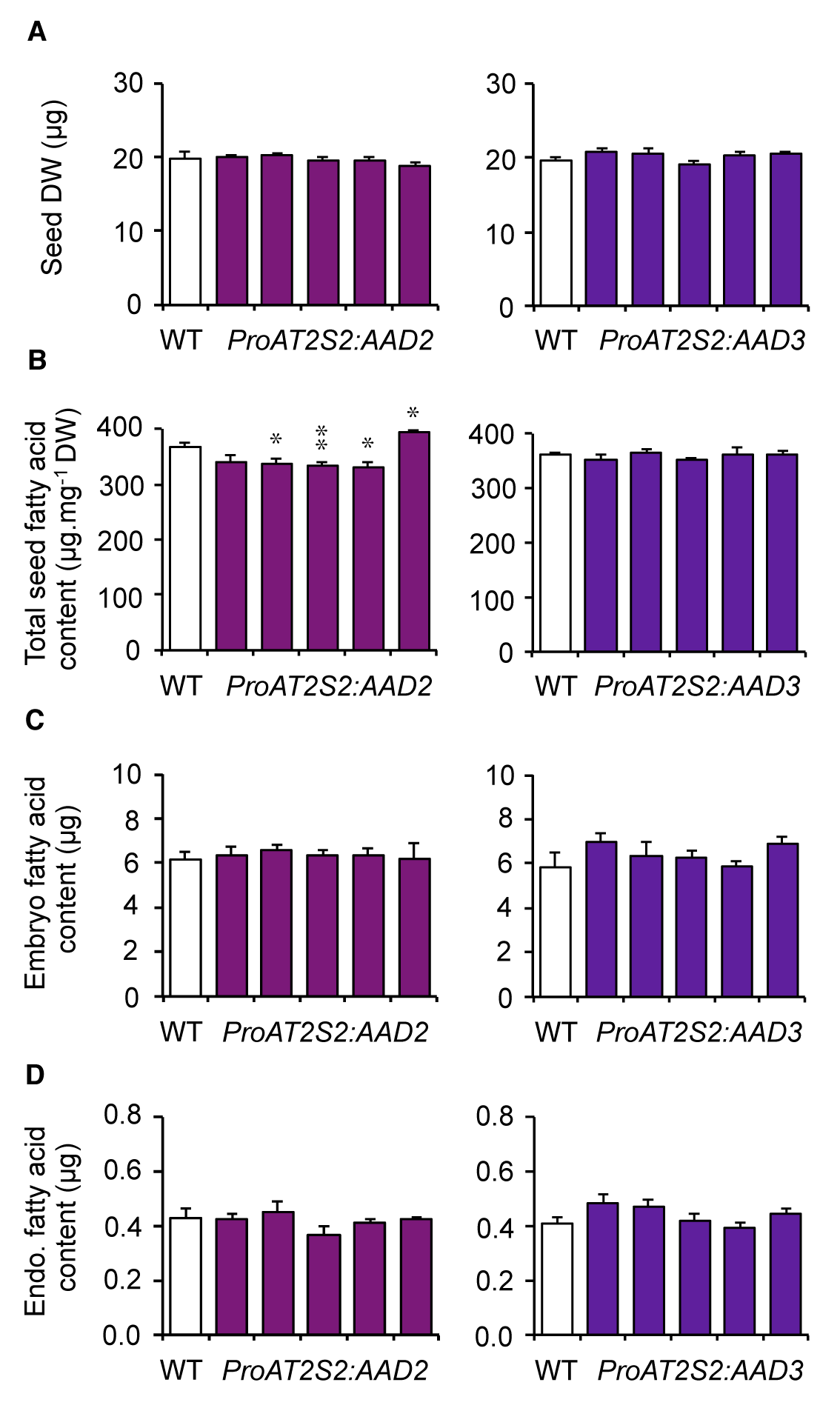

Supplement: S4 Fig — The five independent ProAT2S2:AAD2 transformants considered, T2R3, T19R1, T7R3, T17R1, and T13R8 (in order from left to right) and the five independent ProAT2S2:AAD3 transformants considered, T7R1, T19R5, T16R1, T20R5, and T13R2 (in order from left to right), are always displayed in the same order in the graphs in the figure. (A) Mature seed dry weight. (B) Total fatty acid content of mature dry seeds, expressed in μg.mg-1 DW. (C) Total seed fatty acid content of embryos dissected from mature dry seeds. (D) Total seed fatty acid content of endosperm fractions dissected from mature dry seeds. Values are the means and SE of five replicates carried out on batches of 20 individuals from five plants. Asterisks indicate significant differences from the wild type according to t-test at **P<0.01 and *P<0.05, respectively. (TIF) [file pone.0192156.s004.tif]
