# Supplementary material for: Overexpression of MYB115, AAD2, or AAD3 in Arabidopsis thaliana seeds yields contrasting omega-7 contents
Source: PLoS One. 2018 Jan 30;13(1):e0192156. doi: 10.1371/journal.pone.0192156 (PMC5790276; doi:10.1371/journal.pone.0192156)
Supplement: S1 Table — (PDF) [file pone.0192156.s005.pdf]

S1 Table. Primers used for construct preparation

Construct	Sequence cloned	Primer	Primer sequence (5'→3')
ProAT2S2:uidA	*Beta-glucuronidase* (cDNA)	Forward	GGGGACAAGTTTGTACAAAAAAGCAGGCTTCATGTTACGTCCTGTAGAAACCCC
		Reverse	GGGGACCACTTTGTACAAGAAAGCTGGGTCTCATTGTTTGCCTCCCTGC
ProAT2S2:MYB115	*At4g40360* (cDNA)	Forward	GGGGACAAGTTTGTACAAAAAAGCAGGCTTCATGTATCACCAAAATCTGATTTC
		Reverse	GGGGACCACTTTGTACAAGAAAGCTGGGTCTTAATTCCAACCATTCATGAGC
ProAT2S2:AAD2	*At3g02610* (cDNA)	Forward	GGGGACAACTTTGTATACAAAAGTTGTAATGAAGATGGCTCTTCTCTTG
		Reverse	GGGGACCACTTTGTACAAGAAAGCTGGGTCATGTGTCGTTTTATAGTTCAACTTC
ProAT2S2:AAD3	*At5g16230* (cDNA)	Forward	GGGGACAACTTTGTATACAAAAGTTGTAATGTCGATGGCTT
		Reverse	GGGGACCACTTTGTACAAGAAAGCTGGGTCATCTATCTATAGTTTCACATCTCTACC

Sequences in blue denote DNA recombination sequences (*att* sites).
