# Supplementary material for: Overexpression of MYB115, AAD2, or AAD3 in Arabidopsis thaliana seeds yields contrasting omega-7 contents
Source: PLoS One. 2018 Jan 30;13(1):e0192156. doi: 10.1371/journal.pone.0192156 (PMC5790276; doi:10.1371/journal.pone.0192156)
Supplement: S2 Table — (PDF) [file pone.0192156.s006.pdf]

S2 Table. Primers used for quantitative RT-PCR.

Gene	AGI	Forward primer (5'→3')	Reverse primer (5'→3')
AAD2	At3g02610	QuantiTect Primer Assay (Qiagen) QT00769846	
AAD3	At5g16230	QuantiTect Primer Assay (Qiagen) QT00830564	
AT2S2	At4g27150	CCAGACCACCATCCCTTTCT	CGATGAGGTGTGGTGTTAACA
EF1alphaA4	At5g60390	CTGGAGGTTTTGAGGCTGGTAT	CCAAGGGTGAAAGCAAGAAGA
MYB115	At5g40360	QuantiTect Primer Assay (Qiagen) QT00735623	
