# Supplementary material for: Overexpression of MYB115, AAD2, or AAD3 in Arabidopsis thaliana seeds yields contrasting omega-7 contents
Source: PLoS One. 2018 Jan 30;13(1):e0192156. doi: 10.1371/journal.pone.0192156 (PMC5790276; doi:10.1371/journal.pone.0192156)
Supplement: S3 Table — (PDF) [file pone.0192156.s007.pdf]

**S3 Table. At4g27140/*AT2S2* promoter sequence (5'→3').**

ACGAATCATGTACAATTGTGGATTTGAGATTAAGAATCCATCTGAGAATAGTATACCATTTGTGATTTGTTTATGA
ATGGTATGACACCATAAACGACTATACTACAGTTTGAGATTCACTGATCACTCATAGATTTTTGAATAATTCATTT
GAAAATGATTTGAGATTTTTTTTTTTAAGTGAAATTTTAGTTATTTTGAATTGTTTTTTTTTCTTGTAAAGGGGTA
TAATCCATATTGAAATTCGACCGATACTCAGTCGATAGCCTCTTCACCGAATTTGCTAATGGACCTTAGCCAAGCC
GGAGTTAAAAAAATTTACCGGATAAAAATTGAACCAAAGAGAGAACAAACTGAGACAATGCCCGAAATATATTACT
AGACATTCGCACC**GCATG**TTACAAATTAAACGGACATCGAAAACTACTACATACATACTCAACCTTTTTTATAAAC
TTTTATAGCCATTTTTTGCCAATTTATCTATAACGTAATACTATGCATTCCCTCATTTACATATTTTTTAACATTG
TTCAAAGGGTTGTGTGCCCCGTGTTAATATAGAGTCATTTTCTAACATCTACAATTCAGATTAAGCTCCCAAACAT
ATGTTCCAATTGCAATATACCACCAAATTAAAAGATTGACTCTCACATACCCCATTAATTGAAACCAAATGAACAA
AAACGTTCATAAGATATTAAGATGT**CACGT**CAGAA**CATG**ATCTACAAATGACACATAA**CATGCA**GACGCGGAGACG
CGGAGGGCCGGTGTTGTTCGTCACTTGTCACTCTCTTCCAACACCTAATCCAGACAACAACCTAAGATCTTCACTC
TCGCACACACACGACA**CATGCA**TTCTTA**CACGTG**ATCGC**CATGCA**ATCTCCTTTCTCACCTATAAAACTAACTCTT
CACTTCACTCTTTACTCAAACCAAAACTCATCATCACAAACGAGTAAGAATACAAACACAAATAGCAAAAAA.**ATG**

**RY-like** element containing the CATG core common to the consensus binding sites of the B3-domain transcription factors LEC2, ABI3, and FUS3 (Baud et al., 2016).

**Gbox-like** element.
