# Supplementary material for: Overexpression of MYB115, AAD2, or AAD3 in Arabidopsis thaliana seeds yields contrasting omega-7 contents
Source: PLoS One. 2018 Jan 30;13(1):e0192156. doi: 10.1371/journal.pone.0192156 (PMC5790276; doi:10.1371/journal.pone.0192156)
Supplement: S5 Table — (PDF) [file pone.0192156.s009.pdf]

S5 Table. Total fatty acid composition (in mol%) of endosperm fractions dissected from seeds of engineered lines of *A. thaliana*.

		ProAT2S2:MYB115				
	**Wild type**	**TCR4**	**TAR3**	**TXR2**	**TGR5**	**TJR2**
C16:0	8.25 ± 0.18	7.82 ± 0.06	7.29 ± 0.12	7.54 ± 0.13	7.80 ± 0.16	7.68 ± 0.21
C16:1 (omega-7)	1.77 ± 0.02	1.86 ± 0.02	1.77 ± 0.04	1.80 ± 0.03	2.01 ± 0.03	1.86 ± 0.06
C18:0	3.50 ± 0.13	3.49 ± 0.11	3.16 ± 0.07	3.15 ± 0.11	3.24 ± 0.09	3.29 ± 0.26
C18:1 (omega-9)	9.88 ± 0.31	9.65 ± 0.18	9.54 ± 0.19	9.07 ± 0.18	9.06 ± 0.21	9.05 ± 0.45
C18:1 (omega-7)	6.65 ± 0.22	6.61 ± 0.10	6.69 ± 0.12	6.35 ± 0.15	6.72 ± 0.11	6.38 ± 0.11
C18:2	25.33 ± 0.29	25.71 ± 0.35	25.17 ± 0.18	25.09 ± 0.27	25.32 ± 0.19	25.12 ± 0.45
C18:3	10.30 ± 0.43	10.33 ± 0.13	9.78 ± 0.44	9.72 ± 0.15	9.64 ± 0.12	10.15 ± 0.33
C20:0	2.51 ± 0.04	2.86 ± 0.05	2.83 ± 0.01	2.70 ± 0.07	2.85 ± 0.04	2.82 ± 0.05
C20:1 (omega-9)	9.51 ± 0.05	9.85 ± 0.11	10.08 ± 0.44	9.37 ± 0.21	9.03 ± 0.13	9.33 ± 0.29
C20:1 (omega-7)	12.68 ± 0.33	13.67 ± 0.55	14.40 ± 0.61	13.55 ± 0.43	14.95 ± 0.08	14.02 ± 0.50
C20:2	1.50 ± 0.03	1.74 ± 0.03	1.66 ± 0.04	1.87 ± 0.17	1.65 ± 0.06	1.68 ± 0.06
C22:0	2.87 ± 0.18	2.16 ± 0.40	2.63 ± 0.13	3.33 ± 0.33	2.84 ± 0.22	2.88 ± 0.26
C22:1 (omega-9)	5.26 ± 0.31	4.24 ± 0.60	4.99 ± 0.36	6.46 ± 0.59	4.89 ± 0.43	5.74 ± 0.45
		ProAT2S2:AAD2				
	**Wild type**	**T2R3**	**T19R1**	**T7R3**	**T17R1**	**T13R8**
C16:0	8.32 ± 0.19	6.84 ± 0.13	6.45 ± 0.12	6.86 ± 0.16	6.60 ± 0.08	6.85 ± 0.35
C16:1 (omega-7)	1.80 ± 0.25	2.96 ± 0.04	2.87 ± 0.12	2.71 ± 0.08	2.87 ± 0.07	2.77 ± 0.06
C18:0	4.07 ± 0.28	2.99 ± 0.07	3.11 ± 0.21	3.19 ± 0.15	2.95 ± 0.07	3.46 ± 0.37
C18:1 (omega-9)	10.23 ± 0.21	7.34 ± 0.31	7.37 ± 0.42	7.53 ± 0.27	6.75 ± 0.19	7.28 ± 0.13
C18:1 (omega-7)	6.83 ± 0.27	13.45 ± 0.41	13.78 ± 0.36	12.61 ± 0.39	13.18 ± 0.44	13.58 ± 0.14
C18:2	24.37 ± 0.91	21.81 ± 0.32	21.17 ± 0.50	20.83 ± 0.56	22.29 ± 0.15	20.92 ± 0.24
C18:3	10.15 ± 0.97	9.41 ± 0.36	9.00 ± 0.37	8.47 ± 0.46	9.67 ± 0.23	9.42 ± 0.21
C20:0	2.94 ± 0.22	2.02 ± 0.03	2.17 ± 0.13	2.33 ± 0.17	2.06 ± 0.03	1.98 ± 0.06
C20:1 (omega-9)	10.82 ± 0.32	5.92 ± 0.20	6.45 ± 0.35	6.20 ± 0.25	5.85 ± 0.15	6.01 ± 0.21
C20:1 (omega-7)	15.82 ± 0.32	24.42 ± 0.31	24.91 ± 0.58	26.38 ± 0.41	24.99 ± 0.31	24.58 ± 0.45
C20:2	1.84 ± 0.32	0.99 ± 0.07	0.91 ± 0.06	0.97 ± 0.03	0.92 ± 0.01	1.41 ± 0.45
C22:0	0.47 ± 0.02	0.42 ± 0.01	0.46 ± 0.02	0.45 ± 0.03	0.51 ± 0.01	0.44 ± 0.01
C22:1 (omega-9)	2.34 ± 0.11	1.44 ± 0.10	1.36 ± 0.08	1.47 ± 0.10	1.36 ± 0.02	1.31 ± 0.07
		ProAT2S2:AAD3				
	**Wild type**	**T7R1**	**T19R5**	**T16R1**	**T20R5**	**T13R2**
C16:0	8.01 ± 0.08	6.80 ± 0.20	6.89 ± 0.09	7.43 ± 0.11	6.99 ± 0.08	6.73 ± 0.06
C16:1 (omega-7)	2.04 ± 0.03	2.56 ± 0.04	2.65 ± 0.05	2.62 ± 0.09	2.52 ± 0.04	2.56 ± 0.03
C18:0	3.32 ± 0.10	3.08 ± 0.08	3.09 ± 0.13	3.16 ± 0.07	2.82 ± 0.09	2.95 ± 0.11
C18:1 (omega-9)	9.10 ± 0.09	7.70 ± 0.17	7.93 ± 0.13	8.10 ± 0.26	7.36 ± 0.23	7.39 ± 0.20
C18:1 (omega-7)	7.20 ± 0.20	11.85 ± 0.19	11.36 ± 0.20	12.53 ± 0.55	10.55 ± 0.17	12.32 ± 0.08
C18:2	26.74 ± 0.18	23.41 ± 0.11	23.63 ± 0.24	24.08 ± 0.26	24.31 ± 0.17	23.02 ± 0.13
C18:3	10.95 ± 0.23	10.71 ± 0.28	9.53 ± 0.22	10.81 ± 0.29	10.00 ± 0.29	9.98 ± 0.21
C20:0	2.58 ± 0.04	2.15 ± 0.04	2.19 ± 0.02	1.99 ± 0.09	2.11 ± 0.05	2.09 ± 0.04
C20:1 (omega-9)	9.39 ± 0.12	7.65 ± 0.17	7.36 ± 0.06	7.02 ± 0.40	7.09 ± 0.21	7.15 ± 0.16
C20:1 (omega-7)	16.57 ± 0.25	20.76 ± 0.27	22.03 ± 0.11	19.42 ± 0.24	22.62 ± 0.24	22.63 ± 0.24
C20:2	1.36 ± 0.02	1.05 ± 0.04	1.03 ± 0.04	1.00 ± 0.06	1.06 ± 0.01	0.98 ± 0.02
C22:0	0.60 ± 0.01	0.53 ± 0.02	0.52 ± 0.01	0.52 ± 0.02	0.56 ± 0.01	0.53 ± 0.01
C22:1 (omega-9)	2.15 ± 0.05	1.75 ± 0.05	1.79 ± 0.05	1.31 ± 0.11	2.01 ± 0.08	1.67 ± 0.03

Fatty acid analyses were carried by gas chromatography on dissected endosperm fractions. Values are the means and SE of five replicates carried out on batches of 20 individuals from five plants.
