# Supplementary material for: Overexpression of MYB115, AAD2, or AAD3 in Arabidopsis thaliana seeds yields contrasting omega-7 contents
Source: PLoS One. 2018 Jan 30;13(1):e0192156. doi: 10.1371/journal.pone.0192156 (PMC5790276; doi:10.1371/journal.pone.0192156)
Supplement: S6 Table — (PDF) [file pone.0192156.s010.pdf]

S6 Table. Total fatty acid composition (in mol%) of embryos dissected from seeds of engineered lines of *A. thaliana*.

		ProAT2S2:MYB115				
	**Wild type**	**TCR4**	**TAR3**	**TXR2**	**TGR5**	**TJR2**
C16:0	9.08 ± 0.06	7.46 ± 0.08	7.37 ± 0.03	7.49 ± 0.10	7.51 ± 0.06	7.25 ± 0.10
C16:1 (omega-7)	0.22 ± 0.00	0.82 ± 0.02	0.84 ± 0.02	0.84 ± 0.02	0.78 ± 0.01	0.82 ± 0.03
C18:0	3.33 ± 0.04	2.83 ± 0.02	2.85 ± 0.02	2.89 ± 0.03	2.83 ± 0.03	2.72 ± 0.06
C18:1 (omega-9)	11.64 ± 0.11	9.26 ± 0.18	9.64 ± 0.20	9.26 ± 0.24	9.41 ± 0.23	8.76 ± 0.28
C18:1 (omega-7)	1.75 ± 0.02	5.27 ± 0.09	5.63 ± 0.06	5.64 ± 0.16	5.17 ± 0.04	5.48 ± 0.28
C18:2	28.22 ± 0.17	27.73 ± 0.32	28.25 ± 0.12	28.41 ± 0.21	28.07 ± 0.18	27.61 ± 0.19
C18:3	20.74 ± 0.33	17.10 ± 0.27	16.01 ± 0.22	16.19 ± 0.07	16.86 ± 0.15	17.31 ± 0.30
C20:0	1.81 ± 0.04	1.96 ± 0.02	1.96 ± 0.01	1.96 ± 0.03	1.96 ± 0.02	1.92 ± 0.03
C20:1 (omega-9)	16.91 ± 0.14	17.44 ± 0.09	17.34 ± 0.03	17.28 ± 0.12	17.67 ± 0.11	17.51 ± 0.15
C20:1 (omega-7)	0.98 ± 0.02	4.09 ± 0.10	4.29 ± 0.07	4.34 ± 0.11	3.94 ± 0.08	4.42 ± 0.22
C20:2	2.17 ± 0.12	2.38 ± 0.08	2.35 ± 0.09	2.29 ± 0.04	2.38 ± 0.07	2.48 ± 0.09
C22:0	1.09 ± 0.22	1.06 ± 0.17	1.03 ± 0.05	0.95 ± 0.12	0.96 ± 0.13	1.12 ± 0.02
C22:1 (omega-9)	2.05 ± 0.11	2.61 ± 0.02	2.44 ± 0.02	2.46 ± 0.07	2.47 ± 0.04	2.60 ± 0.07

		ProAT2S2:AAD2				
	**Wild type**	**T2R3**	**T19R1**	**T7R3**	**T17R1**	**T13R8**
C16:0	8.04 ± 0.04	4.46 ± 0.10	4.44 ± 0.11	3.83 ± 0.06	3.78 ± 0.20	3.99 ± 0.17
C16:1 (omega-7)	0.10 ± 0.00	3.64 ± 0.12	3.68 ± 0.06	4.25 ± 0.05	4.21 ± 0.09	4.24 ± 0.20
C18:0	2.95 ± 0.02	3.58 ± 0.24	3.45 ± 0.12	3.54 ± 0.11	3.67 ± 0.24	3.48 ± 0.28
C18:1 (omega-9)	12.41 ± 0.33	3.99 ± 0.25	4.42 ± 0.23	3.67 ± 0.17	3.42 ± 0.23	3.51 ± 0.13
C18:1 (omega-7)	2.16 ± 0.03	25.72 ± 0.79	24.67 ± 0.71	29.37 ± 0.30	31.27 ± 0.57	29.46 ± 0.87
C18:2	27.50 ± 0.07	13.76 ± 0.69	15.07 ± 0.47	12.39 ± 0.54	10.80 ± 0.68	11.46 ± 0.04
C18:3	22.17 ± 0.35	18.00 ± 0.78	18.81 ± 0.09	17.52 ± 0.41	16.53 ± 0.72	16.62 ± 0.75
C20:0	1.82 ± 0.03	2.21 ± 0.15	2.14 ± 0.06	2.12 ± 0.10	1.95 ± 0.13	2.10 ± 0.16
C20:1 (omega-9)	19.28 ± 0.10	7.25 ± 0.46	7.55 ± 0.40	5.84 ± 0.12	5.08 ± 0.32	6.31 ± 0.49
C20:1 (omega-7)	0.00 ± 0.00	15.55 ± 0.98	13.86 ± 0.61	16.20 ± 0.93	17.87 ± 1.08	17.08 ± 0.56
C20:2	1.78 ± 0.03	0.92 ± 0.03	0.92 ± 0.01	0.62 ± 0.16	0.66 ± 0.02	0.82 ± 0.06
C22:0	0.24 ± 0.00	0.22 ± 0.06	0.28 ± 0.01	0.22 ± 0.05	0.26 ± 0.01	0.27 ± 0.01
C22:1 (omega-9)	1.54 ± 0.04	0.71 ± 0.02	0.70 ± 0.03	0.42 ± 0.11	0.51 ± 0.04	0.65 ± 0.08

		ProAT2S2:AAD3				
	**Wild type**	**T7R1**	**T19R5**	**T16R1**	**T20R5**	**T13R2**
C16:0	8.09 ± 0.14	5.49 ± 0.07	5.43 ± 0.04	5.77 ± 0.20	5.45 ± 0.10	5.14 ± 0.04
C16:1 (omega-7)	0.14 ± 0.04	2.78 ± 0.09	2.83 ± 0.06	2.64 ± 0.21	2.77 ± 0.04	3.28 ± 0.03
C18:0	2.91 ± 0.05	2.88 ± 0.05	3.00 ± 0.12	2.86 ± 0.04	2.69 ± 0.06	2.71 ± 0.06
C18:1 (omega-9)	12.17 ± 0.25	5.91 ± 0.42	5.84 ± 0.12	6.03 ± 0.51	5.47 ± 0.20	5.06 ± 0.16
C18:1 (omega-7)	2.43 ± 0.36	18.75 ± 0.51	19.25 ± 0.14	18.52 ± 1.47	18.13 ± 0.69	21.77 ± 0.25
C18:2	27.37 ± 0.27	18.70 ± 0.28	18.48 ± 0.17	19.00 ± 0.79	18.82 ± 0.49	16.98 ± 0.14
C18:3	22.32 ± 0.37	20.17 ± 0.19	20.09 ± 0.13	21.08 ± 0.09	20.90 ± 0.12	20.01 ± 0.10
C20:0	1.84 ± 0.04	1.80 ± 0.02	1.81 ± 0.03	1.73 ± 0.03	1.81 ± 0.03	1.72 ± 0.03
C20:1 (omega-9)	18.69 ± 0.49	10.25 ± 0.40	10.09 ± 0.14	10.18 ± 0.78	10.07 ± 0.31	8.87 ± 0.19
C20:1 (omega-7)	0.42 ± 0.42	10.90 ± 0.40	10.91 ± 0.11	9.94 ± 0.83	11.44 ± 0.36	12.33 ± 0.25
C20:2	1.81 ± 0.04	1.16 ± 0.01	1.12 ± 0.01	1.13 ± 0.06	1.20 ± 0.02	1.05 ± 0.01
C22:0	0.24 ± 0.01	0.25 ± 0.00	0.24 ± 0.00	0.24 ± 0.00	0.23 ± 0.00	0.24 ± 0.00
C22:1 (omega-9)	1.58 ± 0.04	0.95 ± 0.02	0.91 ± 0.01	0.88 ± 0.09	1.01 ± 0.01	0.83 ± 0.01

Fatty acid analyses were carried by gas chromatography on dissected embryos. Values are the means and SE of five replicates carried out on batches of 20 individuals from five plants.
